# Allo-immunisation chez les patients polytransfusés avec des concentrés de globules rouges non phénotypés au Centre Hospitalier Universitaire Souro Sanou de Bobo Dioulasso (Burkina Faso)

**DOI:** 10.11604/pamj.2022.41.244.30952

**Published:** 2022-03-24

**Authors:** Yétéma Dieudonné Yonli, Koumpingnin Nébié, Sourabié Yacouba, Alice Kiba, Salam Sawadogo, Serges Mamadou Sawadogo, Yves Traore

**Affiliations:** 1 Centre National de Transfusion Sanguine (CNTS) Ouagadougou, Burkina Faso,; 2 Département des Sciences Fondamentales et Mixtes, Université Joseph Ki-Zerbo, Ouagadougou, Burkina Faso,; 3 Département des Sciences Fondamentales et Mixtes, Université Nazi Boni, Bobo Dioulasso, Burkina Faso,; 4 Centre de Recherche en Sciences Biologiques Alimentaires et Nutritionnelles (CRSBAN), Université Joseph Ki-Zerbo, Ouagadougou, Burkina Faso

**Keywords:** Allo-immunisation, polytransfusés, concentrés de globules rouges, non phénotypés, Burkina Faso, Alloimmunisation, polytransfused, non-phenotyped red blood cells, Burkina Faso

## Abstract

**Introduction:**

au Burkina Faso, la transfusion sanguine est réalisée en respectant uniquement la compatibilité ABO et RHD entre le donneur et le receveur. Une telle pratique comporte des risques d'allo-immunisation, pouvant engendrer des complications cliniques surtout chez les polytransfusés. L´objectif est de déterminer la prevalence et les facteurs associés à l´allo-immunisation chez les patients polytransfusés avec des concentrés de globules rouges non phénotypés au Centre Hospitalier Universitaire Souro Sanou.

**Méthodes:**

nous avons réalisé une étude transversale chez des patients polytransfusés dans les services cliniques du Centre Hospitalier Universitaire Souro Sanou sur une période de 3 mois (Mars à Mai 2019). Chez chacun des 141 patients inclus, 5 ml de sang total ont été prélevés dans un tube Éthylènediaminetétraacétique (EDTA) pour la recherche des anticorps irréguliers. La recherche des anticorps irréguliers a été réalisée par la technique de Coombs indirect en gel filtration.

**Résultats:**

au total, la fréquence de l´allo-immunisation obtenue est de 5,67%. La majorité des anticorps identifiés appartenaient aux systèmes Rhésus et Kell. Nous n´avons pas trouvés de liaison statistiquement significative entre l´âge, le sexe, les antécédents pathologiques, le nombre de poches transfusés et la positivité de la recherche des anticorps irréguliers (p = 0.37, p= 0,75, p = 0.96).

**Conclusion:**

nous avons trouvé que le risque d´allo-immunisation est majeur. Des mesures supplémentaires doivent être prises pour renforcer la sécurité immunologique des transfusions sanguines au Burkina Faso. Nous proposons qu´au Burkina Faso, la réalisation du test de compatibilité à l´anti-globuline soit systématique chez les patients présentant un grand risque d´immunisation.

## Abstract (en)

**Introduction:**

in Burkina Faso, blood transfusion is carried out with only ABO and RHD compatibility between the donor and the recipient. Such a practice carries risks of alloimmunisation, which can lead to clinical complications especially in polytransfused patients. The objective is to determine the prevalence and factors associated with alloimmunisation in polytransfused patients with non-phenotyped red blood cells at Souro Sanou University Hospital.

**Methods:**

we conducted a cross-sectional study in polytransfused patients in the clinical departments of the University Hospital Souro Sanou over a 3-month period (March to May 2019). In each of the 141 patients included, 5 ml of whole blood was collected in an ethylenediaminetetraacetic acid (EDTA) tube for testing for irregular antibodies. Irregular antibody testing was performed using the indirect Coombs gel filtration technical.

**Results:**

in total, the frequency of alloimmunisation obtained was 5.67%. The majority of the antibodies identified belonged to the Rhesus systems and Kell. We found no statistically significant relationship between age, sex, disease history, number of bags transfused and the positivity of the Irregular Antibody test (p = 0.37, p = 0, 75, p = 0.96).

**Conclusion:**

we found that the risk of alloimmunisation is major. Additional measures should be taken to strengthen the immunological safety of transfusions in Burkina Faso. We propose that in Burkina Faso, anti-globulin compatibility testing should be performed systematically in patients with a high risk of immunisation.

## Introduction

Grâce à l´essor technologique, la transfusion sanguine a beaucoup évolué permettant entre autres, d´améliorer la qualité de vie de la population et d´autoriser des actes et des gestes de soins intensifs (chirurgie lourde, transplantation et greffe) [1]. En effet, les concentrés de globules rouges (CGR) sont des produits sanguins labiles de choix de la thérapeutique transfusionnelle chez certains patients polytransfusés, notamment les drépanocytaires, les thalassémiques, les dialysés, et ceux atteints d´hémopathies. Cependant, comme pour tout produit sanguin labile, la transfusion des CGR expose les patients à des risques infectieux et immunologiques. Parmi ces derniers, figure l´allo-immunisation anti-érythrocytaire post-transfusionnelle qui demeure un problème majeur chez les patients dont la survie dépend des transfusions chroniques avec pour corollaire des situations d´impasses transfusionnelles.

Au Burkina Faso, comme dans beaucoup d´autres pays africains, la transfusion sanguine est réalisée uniquement dans un contexte de compatibilité ABO et RHD sans rechercher la compatibilité dans les autres systèmes érythrocytaires tels que les systèmes Rhésus, Kell, Kidd, Duffy et MNSs, dont les antigènes sont aussi immunogènes. Une telle pratique comporte des risques d'allo-immunisation, pouvant engendrer des complications cliniques surtout chez les polytransfusés mais également compromettre l'avenir obstétrical des sujets de sexe féminin au moment de la procréation [1]. Dans le but d´évaluer la situation de l´allo-immunisation au Burkina Faso, plusieurs études ont été conduite auprès de groupes spécifiques comme les femmes en âge de procréer, les enfants de moins de quinze (15) ans, les dialysés et les drépanocytaires. En effet, Bamouni, 2009 dans son étude sur l´évaluation du risque d´allo-immunisation post-transfusionnelles chez les femmes hospitalisées au service de gynéco obstétriques au Centre Hospitalier Universitaire Souro Sanou (CHUSS) a trouvé un taux d´allo-immunisation de 4.2% [2]. De plus, Kafando, 2017 dans une étude sur les cas de transfusion incompatible chez les enfants de 6 mois à 15 ans a trouvé un taux d´allo-immunisation de l´ordre de 4.2% [3]. Aussi, Nébié, 2019 dans une étude chez les patients dialysés et drépanocytaires a trouvé une allo-immunisation globale de 3,2% [4].

Le constat est qu´aucune étude n´a été réalisée pour évaluer la situation de l´allo-immunisation dans toutes les situations de poly-transfusion au Burkina Faso. C´est pour cela nous avons voulu évaluer la prévalence de l´allo-immunisation anti-érythrocytaire dans les différentes situations de poly-transfusions et les facteurs associés à ces allo-immunisation au CHU Souro SANOU, Bobo Dioulasso, Burkina Faso.

## Méthodes

**Conception et cadre de l´étude:** nous avons conduit une étude transversale sur une durée de 3 mois allant de Mars à Mai 2019. Les patients ont été recrutés au sein des différents services cliniques du CHUSS. Les investigations biologiques se sont réalisées au laboratoire d´immunologie du Centre National de Transfusion Sanguine (CNTS) à Ouagadougou. Le CHU-SS est l´un des trois centres hospitaliers universitaires du Burkina Faso. Il constitue un centre national de référence et reçoit outre les malades de la province du Houet, ceux évacués des provinces environnantes. Il a une capacité de 690 lits et est divisé en sept départements. Il est l´unique hôpital de la ville de Bobo Dioulasso. Le CNTS est un centre national de transfusion sanguine à Ouagadougou. Il assure la disponibilité en qualité et en quantité des produits sanguins sur toute l´étendue du territoire à travers ses antennes régionales au nombre de dix.

**Population de l´étude:** la population d´étude est constituée de patients polytransfusés. Les critères d´inclusions c´est d´avoir subi plus de deux (02) séances de transfusions sanguines. Les patients ayant reçu un seul acte de transfusion sanguine ont été exclus de l´étude. Le manque de réactif pour la RAI nous a amené à fixer la taille de l´échantillon à 141 patients. Chez les patients inclus, 5 ml de sang total ont été prélevés au moment de la demande de sang dans un tube avec EDTA dument étiqueté.

**Variables:** pour chaque patient inclus, nous avons identifié les facteurs suivants comme pouvant influencer l´allo-immunisation: l´âge, le sexe, l´état pathologique des patients, le nombre d´unités de CGR transfusés, les antécédents transfusionnels.

**Analyse au laboratoire:** la RAI (recherche d´agglutinines irrégulières) a été réalisée sur chaque prélèvement selon un mode opératoire standard pour le diagnostic de l´allo-immunisation. Pour tous les patients qui ont présenté une allo-immunisation, on a procédé à une identification de l´anticorps. Le dépistage et l´identification étaient réalisés selon la technique de Coombs Indirect ou Test Indirect à l´anti globuline (TIA) sur support de colonne de filtration (Carte gel à l´anti globuline Humaine). C´est un test d´agglutination. Il est utilisé pour détecter les anticorps (Ig G) présents dans le plasma et dirigés contre les globules rouges de patients. Le plasma du patient est incubé avec les globules rouges réactifs; puis le serum de Coombs (anticorps contre les Ig G humaines ou anti- Ig G humaines) est ajouté. Si une agglutination se produit, des anticorps IgG (allo- ou auto- anticorps) sont présents.

**Analyses statistique:** les données ont été saisies et analysées à l'aide du logiciel Epi-info version 7.2.0.1. Le test statistique exact de Fischer a été utilisé pour la comparaison des différentes proportions et la valeur de p < 0,05 a été retenue comme seuil de signification.

**Considérations éthiques:** les patients polytransfusés par les concentrés globulaires de notre étude ont reçu des traitements médicaux adéquats. L´étude a reçu l´approbation du comité d´éthique pour la recherche en santé du Burkina sous le numéro: 2020-08-32. Les participants à l´étude ont donné leurs consentements par écrit après informations.

## Résultats

**Caractéristiques sociodémographiques:** au total, 141 patients ont été inclus dans l´étude. Nous avons noté une prédominance des patients de sexe masculin 105 soit 74,47%. Et les patients de sexe féminin sont de 36 sujets soit 25.53%. L´âge moyen des patients était de 34,90 ± 23,47 ans.

**Immunisation et profils des anticorps:** parmi les 141 patients polytransfusés retenus dans notre étude, 08 ont présenté une situation d´allo-immunisation soit un taux global de 5,67%. Nous avons retrouvé au total 06 allo-anticorps avec 03 situations d´associations d´anticorps. Les allo-anticorps anti K sont les plus représentés 2,12% (Tableau 1).

**Tableau 1:** répartition des allo-anticorps chez les patients

Type d'allo-anticorps	Effectif(n= 8)	Prevalence (%)
Anti E	1	0,70
Anti E et anti lua	1	0,70
Anti E et anti M	1	0,70
Anti K et anti M	1	0,70
Anti K	3	2,12
Anti Kpa1	1	0,70
Total	8	**5,67**

**Caractéristiques des patients allo-immunisés:** la recherche d´agglutinine nous a permis de déterminer les caractéristiques des patients ayant développé des allo-anticorps comme représenté dans le Tableau 2.

**Tableau 2:** caractéristiques des patients ayant développés des allo-anticorps

Allo-anticorps	Sexe	Age moyen (ans)	nombre de poches reçues	Pathologies associés
Anti K et anti M	F	44	2	VIH
Anti K	M	21	2	LAM
Anti K	M	59	4	IRC
Anti K	F	42	3	IRC
Anti E et anti M	M	71	2	VIH
Anti E	M	49	2	Ulcère
Anti Kpa	M	77	4	Adénocarcinome
Anti E et anti lua	M	5	2	Lymphome Burkitt

**Répartition des patients en fonction des facteurs associés à l´allo-immunisation:** a travers une analyse, nous avons croisé le sexe, l´âge, le type de pathologie et le nombre de poche reçues avec la présence d´allo anticorps pour déterminer les facteurs associés à l´allo-immunisation. Nous n´avons pas trouvés de liaison statistiquement significative entre l´âge, le sexe, les antécédents pathologiques, le nombre de poches transfusés et la positivité de la Recherche des Anticorps Irréguliers (p = 0.37, p = 0,75, p = 0.96) (Tableau 3).

**Tableau 3:** répartition des patients en fonction des facteurs associés à l’allo-immunisation

Caractéristiques des patients	Effectif (n=8)	Prevalence des allo-anticorps	P value
**Sexe**			**0,75**
M	6	4,25	
F	2	1,41	
**Age (ans)**			**0,37**
>15	1	0,70	
<15	7	4,96	
**Nombre de poches reçues**			**0,96**
2 à 5 poches	8	5,67	
Plus de 5 poches	0	0,00	
**Situations cliniques**			**0,56**
Cancers	1	0,70	
Intervention chirurgicale	0	0,00	
Drépanocytose	0	0,00	
Hémopathies malignes	1	0,70	
Insuffisance rénale	2	1,41	
Pathologies infectieuses	0	0,00	
Autres	4	2,83	

## Discussion

Nous avons voulu à travers cette étude, déterminer la prévalence et les facteurs associés à l´allo-immunisation chez les patients polytransfusés avec des concentrés de globules rouges non phénotypés au Centre Hospitalier Universitaire Souro Sanou. Au total, la fréquence de l´allo-immunisation obtenue est de 5,67%. La majorité des anticorps identifiés appartenaient aux systèmes Rhésus et Kell. Nous n´avons pas trouvés de liaison statistiquement significative entre l´âge, le sexe, les antécédents pathologiques, le nombre de poche transfusé et la positivité de la recherche des anticorps irréguliers (p = 0.37, p = 0,75, p = 0.96).

Nous avons trouvé un taux global d´immunisation de 5,67%. Ce taux peut être considéré comme assez faible car contrairement à ce qui se passe en occident où la recherche d´agglutinines irrégulières (RAI) fait partie systématiquement des moyens de prévention des complications transfusionnelles, au Burkina Faso on note une absence de précautions particulières préalables aux transfusions érythrocytaires. En effet, seule la compatibilité dans le système ABO et pour l´Ag RHD est respectée avant toute transfusion. Cette situation semble augmenter les risques d´immunisations surtout dans les autres systèmes notamment le RH et le Kel qui sont très immunogènes. Certaines études conduites chez les polytransfusés dans des pays où des mesures pour assurer la compatibilité transfusionnelle sont mises en places et respectées nous rapportent des taux d´immunisations plus importantes. En France, on a enregistré un taux de 50% d´allo-immunisation dans une cohorte de 206 patients drépanocytaires [5]. Aux Etats Unis d´Amérique, Chou et Aygun rapportaient des taux d´immunisation, de l´ordre de 29% à 46% chez des drépanocytaires polytransfusés [6,7]. En Côte d´ivoire, pays voisin du Burkina Faso, Dembélé B *et al*. avaient retrouvé un taux d´allo-immunisation de 14,2% chez 288 patients de profils pathologiques divers c´est-à-dire un profil de patients semblable à notre population d´étude [8]. Cela pourrait être dû au panel utilisé dans notre étude qui est celui destiné en priorité à des populations caucasiennes. Ainsi, certaines situations d´immunisations spécifiques à des antigènes qu´on ne rencontre que dans des populations noires africaines pourraient passer inaperçues. De plus nous n´avons pas pu évaluer les délais entre la dernière transfusion sanguine et l´inclusion. Cette situation pourrait contribuer à une sous-estimation des cas d´immunisation étant donné que, la présence des anticorps peut être faible et passée inaperçue si la transfusion est trop récente. De plus nous n´avons pas pu réaliser le phénotype des patients allo-immunisés et de comparer la spécificité des anticorps trouvés au patrimoine antigéniques des patients. Ce qui pourrait entrainer aussi une sous-estimation des cas d´immunisation.

Par ailleurs, nous n´avons pas trouvé une association significative entre l´allo-immunisation et des facteurs comme l´âge, le sexe, le type d´antécédent pathologique et le nombre de poche de sang reçu. Nos résultats notamment ceux relatif au nombre de poche de sang reçu semblent traduire la réalité. En effet, si la plupart des études suggèrent une association entre le nombre de poche reçu et le risque d´allo-immunisation, dans la nôtre, la totalité des patients allo-immunisés avaient reçu un nombre de poche compris entre 2 et 5 poches de CGR. Cependant, nos résultats nous renseignent que ce ne sont pas uniquement les drépanocytaires et les dialysés beaucoup plus exposés aux produits sanguins qui sont les plus à risque d´allo-immunisation mais tout patient soumis à des transfusions itératives présente un risque d´allo-immunisation. En effet, Kafando *et al*. rapportaient des résultats similaires au notre dans une étude ayant recherché l´allo-immunisation chez 477 enfants transfusés sans compatibilité particulière autre que ABO et RHD au CHU Pédiatriques Charles de Gaulle de Ouagadougou. Ils avaient trouvé un taux d´immunisation de l´ordre 4.2% [3].

**Limites et points forts:** la principale limite est liée au type de panel utilisé pour la RAI. C´est un panel destiné en priorité à des populations caucasiennes. L´absence du phénotypage des patients allo-immunisés. La majeure partie des études rencontrées dans la littérature se sont surtout focalisées dans les services de pédiatrie, d´hématologie ou de néphrologie. Ces services sont réputés accueillir des patients tels que les drépanocytaires, les dialysés, des patients souffrant d´hémopathies malignes, sujets donc à des transfusions itératives. Dans notre contexte nous avons élargi cette étude à toutes les pathologies qui exposent certains patients à de multiples transfusions sanguines.

## Conclusion

Le taux d´allo-immunisation obtenu nous a semblé assez faible au regard des pratiques transfusionnelles dans le pays. Tout de même, nous avons trouvé que le risque d´allo-immunisation est majeur. Des mesures supplémentaires doivent être prises pour renforcer la sécurité immunologique des transfusions sanguines au Burkina Faso. Nous proposons qu´au Burkina Faso, la réalisation du test de compatibilité à l´anti-globuline soit systématique chez les patients présentant un grand risque d´immunisation.

### Etat des connaissances sur le sujet

- La Recherche systématique des anticorps irréguliers et la sélection de concentrés de globules rouges phénotypés dans les systèmes Rhésus et Kell sont préconisées par plusieurs pays pour la sécurité transfusionnelle;
- Cette recherche n´est cependant pas systématique dans les environnements à ressources limitées;
- Les études sur l´allo-immunisation chez les patients polytransfusés avec des concentrés de globules rouges non phénotypés sont partielles.

### Contribution de notre étude à la connaissance

- La réalisation du test de compatibilité systématique à l´anti globuline chez les patients présentant le plus grand risque d´immunisation (drépanocytaires, dialysés, patients souffrant d´hémopathies malignes etc) doit être une solution à mettre place dans l´immédiat;
- L´âge, le sexe, les antécédents pathologiques, le nombre de poches transfusés ne sont pas sont associés à la positivité de la recherche des anticorps irréguliers.

## Remerciements

Nous remercions les patients du CHU Souro SANOU.qui ont bien voulu participer à cette étude. Nous tenons à remercier la Directrice Générale du Centre National de Transfusion Sanguine (CNTS) Ouagadougou, Burkina pour sa contribution dans l´obtention des réactifs de laboratoire. Des remerciements particuliers au Pr Yves TRAORE de l´Université Joseph Ki-Zerbo, UFR/SVT, Ouagadougou, Burkina Faso pour sa lecture critique de l´article.
